# Fe-based superconducting transition temperature modeling by machine learning: A computer science method

**DOI:** 10.1371/journal.pone.0255823

**Published:** 2021-08-06

**Authors:** Zhiyuan Hu

**Affiliations:** China University of Mining and Technology Beijing, Beijing, China; Mohanlal Sukhadia University, INDIA

## Abstract

Searching for new high temperature superconductors has long been a key research issue. Fe-based superconductors attract researchers’ attention due to their high transition temperature, strong irreversibility field, and excellent crystallographic symmetry. By using doping methods and dopant levels, different types of new Fe-based superconductors are synthesized. The transition temperature is a key indicator to measure whether new superconductors are high temperature superconductors. However, the condition for measuring transition temperature are strict, and the measurement process is dangerous. There is a strong relationship between the lattice parameters and the transition temperature of Fe-based superconductors. To avoid the difficulties in measuring transition temperature, in this paper, we adopt a machine learning method to build a model based on the lattice parameters to predict the transition temperature of Fe-based superconductors. The model results are in accordance with available transition temperatures, showing 91.181% accuracy. Therefore, we can use the proposed model to predict unknown transition temperatures of Fe-based superconductors.

## 1 Introduction

Superconductors with the zero resistance and the Meissner effect have significant practical application [1]. The best known application is in the Magnetic Resonance Imaging (MRI) systems widely employed by health care professionals for detailed internal body imaging. Other prominent applications include the magnetically levitated trains without friction and electrical power transmission with no energy loss [2–5]. However, superconductors have superconductivity only at or below their transition temperature [6], which hold back the wide spread application of superconductors.

Researchers have been conducting an extensive search for novel superconductors, especially those with high transition temperature. High temperature superconductors such as cuprate superconductors containing CuO_2_ planes [7–10], MgB_2_ [11], hydride superconductors under extreme pressure [12–18], and Fe-based superconductors [19]. In particular, Fe-based superconductors have high transition temperature next to cuprates, an upper critical field above 50T, a relatively strong irreversibility field, and a high crystallographic symmetry [20], which attract the attention of researchers. In the process of exploring the influencing factors of Fe-based superconducting transition temperature, a strong relationship between the transition temperature and the lattice parameters is found [21–26]. According to composition and crystal structure, Fe-based superconductors are divided into four categories, including ReFeAsO (Re = rare earth elements) (1111 system), AFe_2_As_2_ (A = K, Sr, Ba, etc.) (122 system), LiFeAs (111 system), and FeSe (11 system).

At present, one of the main research directions of Fe-based superconductors is to improve their transition temperature via various doping methods and dopant levels [27,28]. The transition temperature is a key indicator to measure whether new superconductors are high temperature superconductors. However, the measurement of the transition temperature needs high precision devices including temperature controllers, constant current sources, and voltmeters, etc. These conditions cannot be achieved by ordinary laboratories. Meantime, it is necessary to artificially operate liquid nitrogen (77K) in the measurement process, and there are certain security risks. In addition, it mainly depends on liquid helium (4.2K) as refrigerant for superconductors that have strict temperature requirements. Because the equipment for liquefied helium is very complicated, and the liquid helium (4.2K) temperature is close to the absolute zero, the measurement of the transition temperature is very difficult.

Machine Learning (ML) is one branch of artificial intelligence while it is currently in the process of growth and evolution and is an active field in data science. One of the application of ML is data mining. In past decades, algorithms and theories corresponding to ML have had many advances, including the provision of useful data and robust computing infrastructures. Data mining is now rapidly applied to superconducting material science. Examples include using a Gaussian regression algorithm to predict physical parameters of superconductors [29–38]; using support vector regression [39], random forest algorithm [40], and XGBoost model [41] to predict high temperature superconductor candidates; and using GMDH-type neural network [42] to predict hysteresis loops of superconductors. The BP algorithm has excellent complex pattern classification and multi-dimensional function mapping capabilities, and it is applied in function fitting, data analysis and prediction. To avoid the strict measurement conditions and risk factors of the transition temperature measurement process, in this paper, we adopt a machine learning method to build a model based on the lattice parameters to predict the transition temperature of Fe-based superconductors.

## 2 Computational method

### 2.1 Description of BP algorithm

**Fig 1:**
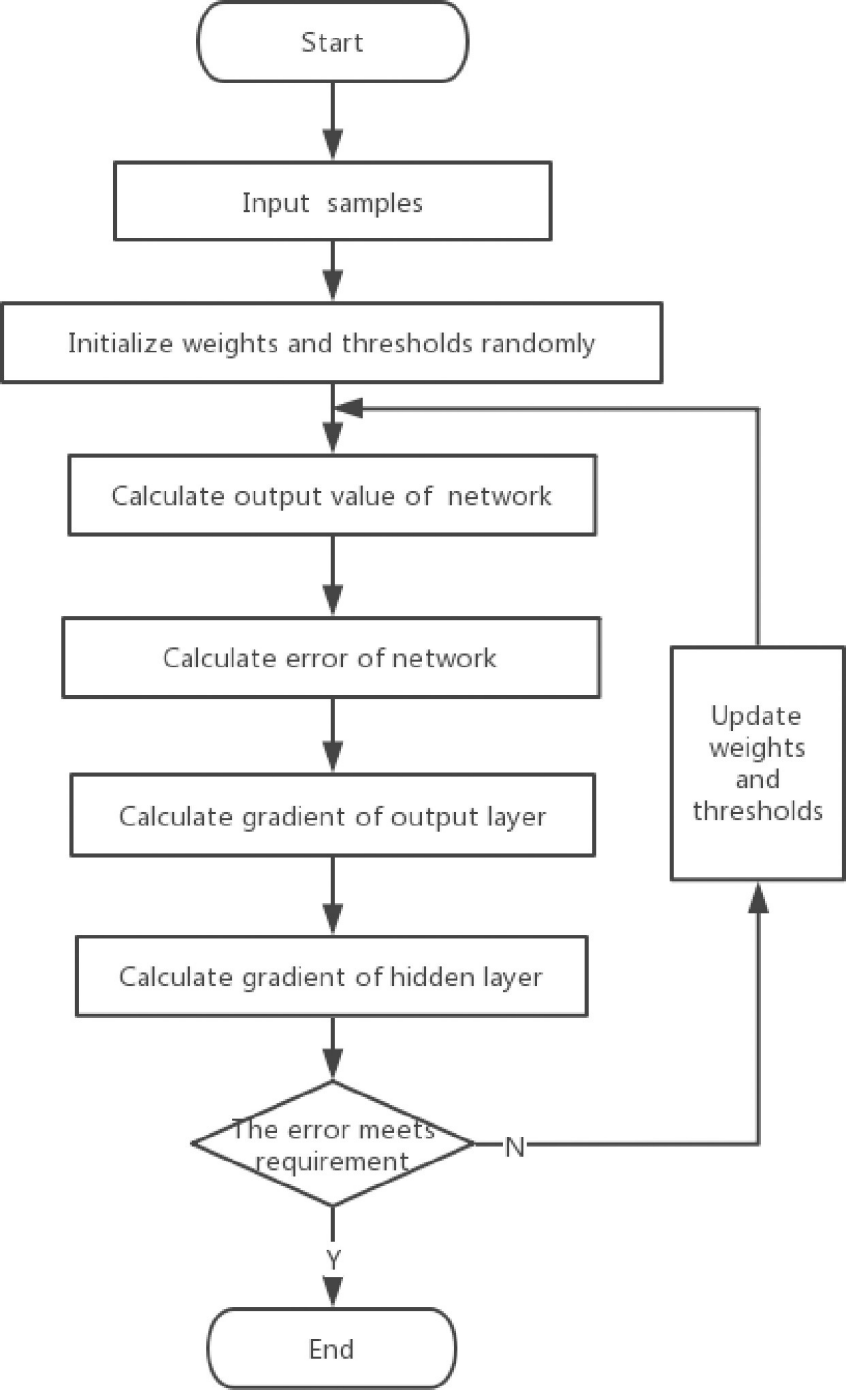
BP algorithm flowchart.

The BP algorithm is a type of error back propagation algorithm. Error back propagation consists of forward propagation and feedback based on error signals. The forward propagation direction is input layer→hidden layer→output layer. The state of each layer of neurons only affects the state of the next layer of neurons. If the expected value is not obtained in the output layer, error signals will propagate back. The back propagation direction is output layer→hidden layer→input layer. By adjusting the weights and thresholds of each layer, the error will decrease along the negative gradient direction. The weights and thresholds are continuously iterated until the error meets the precision requirements. The algorithm process is shown in (Fig 1).

### 2.2 Calculation of BP algorithm

**Fig 2:**
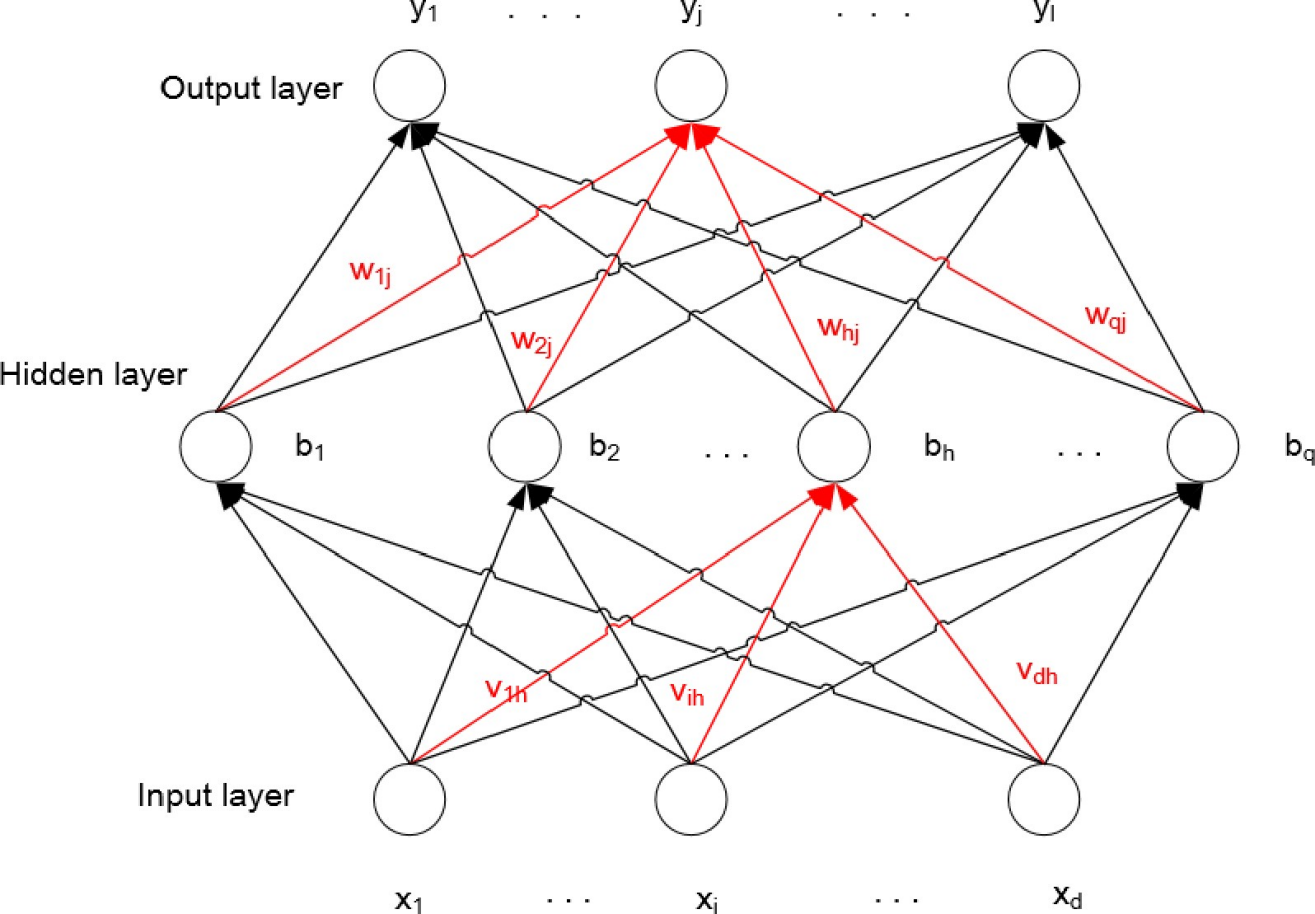
Network topology diagram.

Assume a three-layer network with d-dimension input, l-dimension output, and q-dimension hidden layer, as shown in (Fig 2). In the network, the threshold of the j−th neuron in the output layer is *θ*_*j*_, the threshold of the h−th neuron in the hidden layer is *γ*_*h*_, the weight between the i−th neuron in the input layer and the h−th neuron in the hidden layer is *v*_*ih*_, and the weight between the h−th neuron in the hidden layer and the j−th neuron in the output layer is *w*_*hj*_.

The input of the h−th neuron in the hidden layer is:

$$\alpha_{h}=\sum_{i=1}^{d}v_{ih}x_{i}.$$ (1)

The input of the j−th neuron in the output layer is:

$$\beta_{j}=\sum_{h=1}^{q}w_{hj}b_{h},$$ (2)

where, *b*_*h*_ is the output of the h−th neuron in the hidden layer.

For a training example (x_k_, y_k_), the output of a neuron is ${\tilde{y}}_{k}{=(\tilde{y}}_{1}^{k},{\tilde{y}}_{2}^{k},\ldots ,{\tilde{y}}_{l}^{k})$,

$${\tilde{y}}_{j}^{k}=f(\beta_{j}-\theta_{j}),$$ (3)

where, f() is an activation function.

The mean square error is:

$$E_{k}=\frac{1}{2}{\left({\tilde{y}}_{j}^{k}-y_{j}^{k}\right)}^{2},$$ (4)

where, $y_{j}^{k}$ is the actual value.

BP algorithm is an iterative algorithm, and the updating formula of parameter v is:

v←v+Δv. (5)

The weight *w*_*hj*_ between hidden layer and output layer is:

$$\Delta w_{hj}=-\eta \frac{\partial E_{k}}{\partial w_{hj}},$$ (6)

where, η is the learning rate.

According to the chain rule:

$$\frac{\partial E_{k}}{\partial w_{hj}}=\frac{\partial E_{k}}{{\partial \tilde{y}}_{j}^{k}}∙\frac{{\partial \tilde{y}}_{j}^{k}}{\partial \beta_{j}}∙\frac{\partial \beta_{j}}{\partial w_{hj}}.$$ (7)

According to the definition of *β*_*j*_:

$$\frac{\partial \beta_{j}}{\partial w_{hj}}=b_{h}.$$ (8)

According to formulas (3) and (4):

$$g_{j}=-\frac{\partial E_{k}}{{\partial \tilde{y}}_{j}^{k}}∙\frac{{\partial \tilde{y}}_{j}^{k}}{\partial \beta_{j}}=-({\tilde{y}}_{j}^{k}-y_{j}^{k})f^{\prime }(\beta_{j}-\theta_{j}).$$ (9)

The updating formula of weights and thresholds is:

$$\Delta w_{hj}=\eta g_{j}b_{h}.$$ (10)

$$\Delta \theta_{j}=-\beta g_{j}.$$ (11)

$$\Delta v_{ih}=\eta e_{h}x_{i}.$$ (12)

$$\Delta \gamma_{h}=-\eta e_{h}.$$ (13)

In the formulas (12) and (13):

$$e_{h}=-\frac{\partial E_{K}}{\partial b_{h}}∙\frac{\partial b_{h}}{\partial \alpha_{h}}=b_{h}(1-b_{h})\sum_{j=1}^{l}w_{hj}g_{j}.$$ (14)

By continuously iterating the weights *w*_*hj*_, and *v*_*ih*_, as well as the thresholds *θ*_*j*_, and *γ*_*h*_, the accuracy of the network will continue to improve. The performance of the trained network is evaluated by the mean absolute error (MAE), the root mean square error (RMSE), and the correlation coefficient (CC).

## 3 Data description

**Fig 3:**
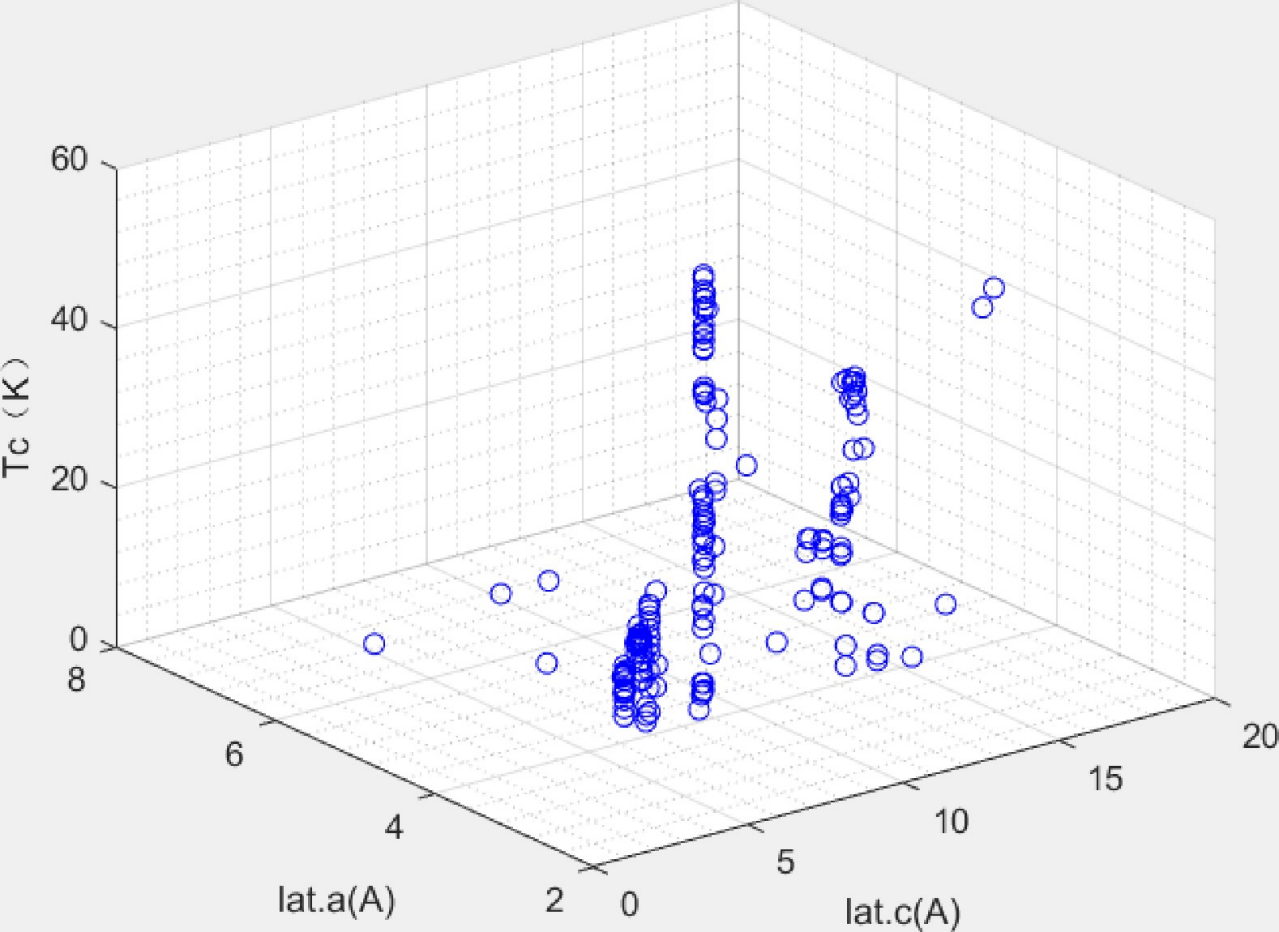
Transition temperature, Tc (K), and lattice parameters, lat.a (A) and lat.c (A).

Data were obtained from Japan’s National Institute for Materials Science (NIMS) at http://supercon.nims.go.jp/index_en.html. After data processing, 203 sets of data were collected. To strengthen data relevance, the data only contained four representative types of Fe-based superconductors, namely 11, 111, 122, and 1111. The space distribution characteristic of data between the transition temperature and the lattice parameters is displayed in (Fig 3). The data were roughly divided into the 4 groups, corresponding to the 4 types of Fe-based superconductors. Each group has a certain degree of discreteness and a non-linear relationship, which meets the modeling requirements.

**Fig 4:**
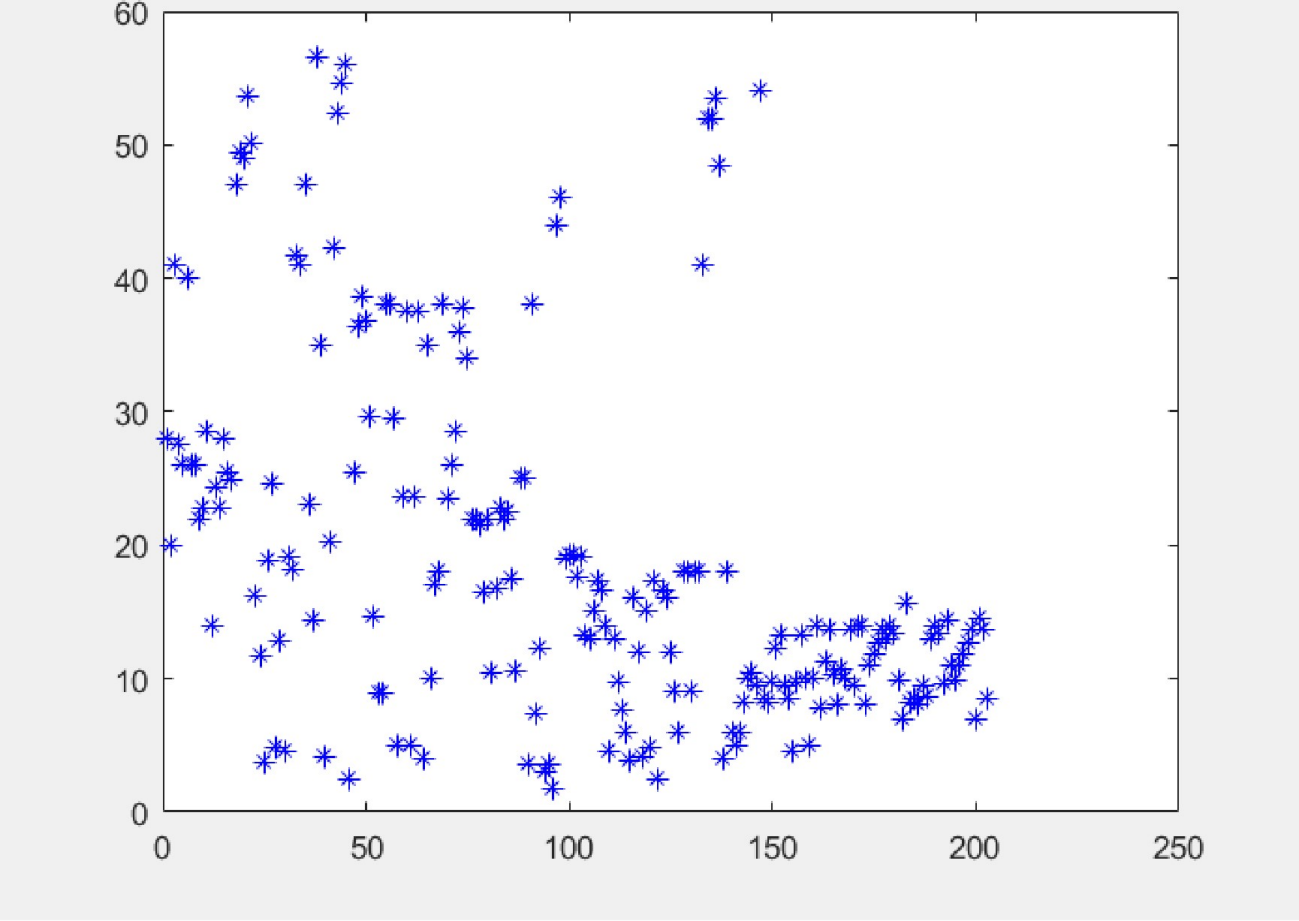
Transition temperature distribution of samples.

The visualization of transition temperature is shown in (Fig 4), which is discrete, and there is no aggregation. The data are distributed among 0–60 K, which is consistent with the transition temperature range of Fe-based superconductors. Statistical analysis of the transition temperature—including maximum, minimum, mean, variance, standard deviation (std), range, median, coefficient of variation, and skewness—is presented in (Table 1). The coefficient of variation is 70.03%, indicating the transition temperature has good dispersion. The skewness is greater than zero, indicating the data greater than the mean value are more scattered than the data less than the mean value.

**Table 1:** Statistical analysis of Tc in (Fig 4).

Parameter	Data
**Maximum**	56.5000
**Minimum**	1.8000
**Mean**	19.3666
**Variance**	183.9457
**Std**	13.5627
**Range**	54.7000
**Median**	14.4000
**Coefficient of variation**	0.7003
**Skewness**	1.1113

## 4 Result and discussion

### 4.1 Model accuracy

**Fig 5:**
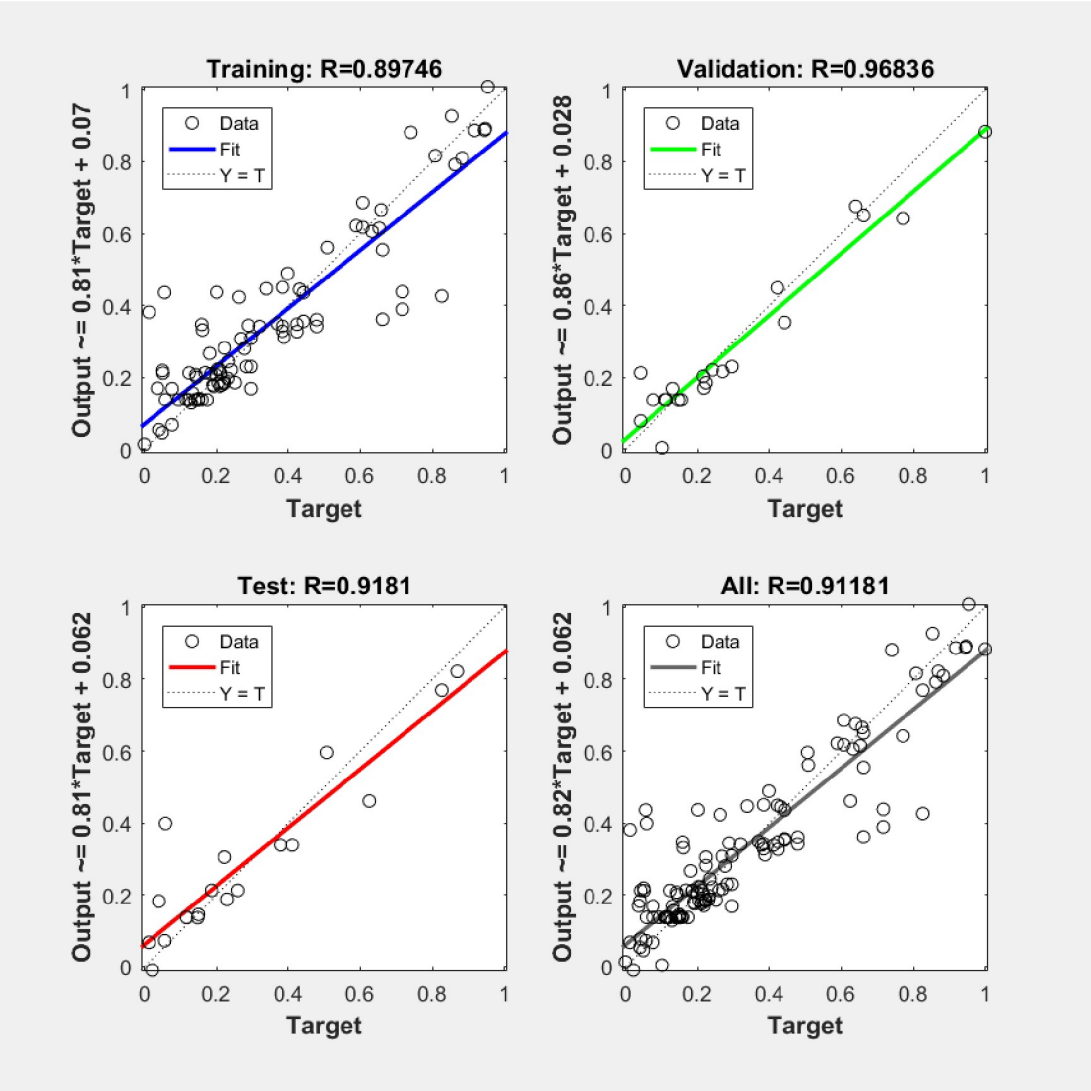
Regression analysis.

This paper divides the 203 sets of data into 2/3 training data and 1/3 testing data at random, and trains the model. The regression analysis between the actual transition temperature and the estimated transition temperature in the course of training the model are presented in (Fig 5) with accuracy of 91.181%. It shows a reasonable accuracy and powerful generalization. The performance of the model is shown in (Table 2). The MAE and CC are 0.47265, and 85.44%, respectively, representing closely matching performance and good prediction performance.

**Table 2:** Performance of the model.

Parameter	Data
**MAE**	0.47265
**RMSE**	7.6863
**CC**	0.8544

### 4.2 Model stability

To further estimate on the prediction stability, the model performance measures through the 5 predictions for observation in (Table 3). It is found that all predictions generally maintain high accuracy from the training sample. The std of the MAE, RMSE, and accuracy are 0.0245, 0.05162, and 0.7057%, meaning that prediction errors are in a controllable range and that the model has a good prediction stability.

**Table 3:** Model evaluation.

Parameter	MAE	RMSE	Accuracy
**1st**	0.47265	7.6863	91.181%
**2st**	0.47845	8.4063	91.296%
**3st**	0.50231	7.1271	90.382%
**4st**	0.47675	8.3406	89.428%
**5st**	0.42716	8.4370	91.131%
**Minimum**	0.42716	7.1271	89.428%
**Maximum**	0.50231	8.4370	91.296%
**Mean**	0.471464	7.99946	90.6836%
**Median**	0.47675	8.3406	91.131%
**Std**	0.0245	0.5162	0.7057%

### 4.3 Comparisons with previous studies

In (Table 4), the performance of our BP model is compared with that based on two other models, the RF (Random Forest) [43] and the MLR (Multi-variable Linear Regression Regression) [44], in previous studies. It is found that our BP model has a optimal performance in terms of the CC and accuracy. In addition, our BP model is more straightforward from the perspective of computations and implementations than the others.

**Table 4:** Model performance comparisons.

Model	CC	Accuracy
**RF**	0.82	88.26%
**MLR**	0.84	88%
**BP**	0.8544	91.181%

### 4.4 Fe-based superconductors prediction

**Fig 6:**
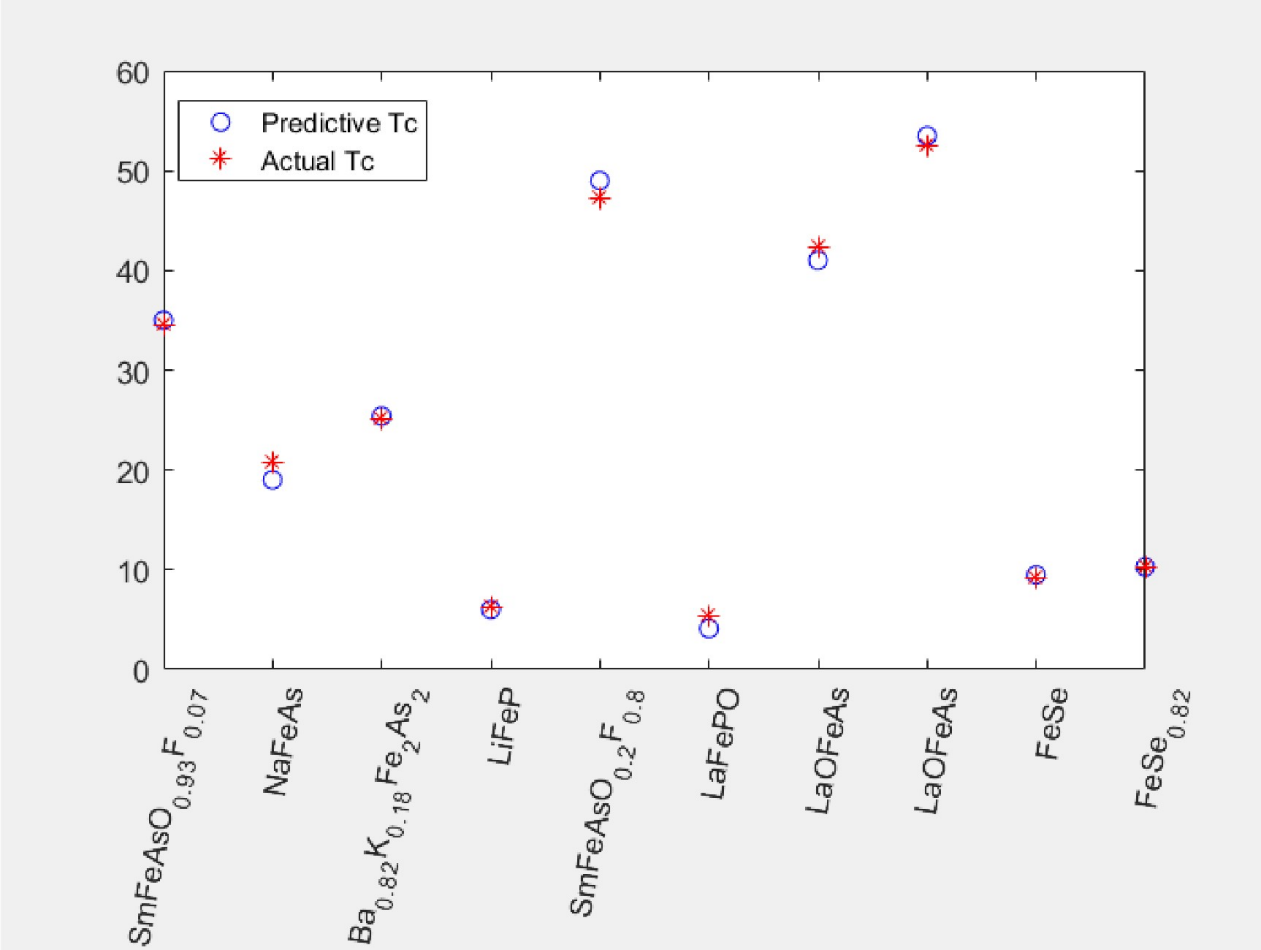
Comparison of predictive value and actual value.

In order to identify the feasibility and validity of the new model, 10 Fe-based superconductors include the four kinds of Fe-based superconductors, whose transition temperature values are in a range of 4.1–53.5K, were selected from the literature [45–50] that are not included in the trained model as the data. We input the lattice parameters of every Fe-based superconductor into the model, and obtain the corresponding predictive transition temperature. The results are presented in (Table 5) and the visualization is shown in (Fig 6). The superconductors NaFeAs, SmFeAsO_0.2_F_0.8_, LaFePO, and LaOFeAs have a slightly larger error (1-2K), and the superconductors SmFeAsO_0.93_F_0.07_, Ba_0.82_K_0.18_Fe_2_As_2_, LiFeP, FeSe, and FeSe_0.82_ have a good accuracy (0.2–0.5K). The result shows that the model we build achieves an acceptable accuracy and we can measure the transition temperature of Fe-based superconductors based on the lattice parameters.

**Table 5:** Model prediction result.

Elements	Lat.a	Lat.c	Tc	Prediction	References
**SmFeAsO**_**0.93**_**F**_**0.07**_	3.393	8.482	35.0	34.4591	[45]
**NaFeAs**	3.928	6.364	19.0	20.6925	[45]
**Ba**_**0.82**_**K**_**0.18**_**Fe**_**2**_**As**_**2**_	3.937	13.155	25.4	25.0637	[46]
**LiFeP**	3.692	6.031	6.0	6.2621	[47]
**SmFeAsO**_**0.2**_**F**_**0.8**_	3.931	8.477	49.0	47.1806	[48]
**LaFePO**	3.962	8.511	4.1	5.2642	[49]
**LaOFeAs**	4.035	8.740	41.0	42.4102	[49]
**LaOFeAs**	4.035	8.435	53.5	52.4721	[49]
**FeSe**	3.770	5.521	9.5	9.1126	[50]
**FeSe**_**0.82**_	3.770	5.510	10.3	10.1835	[50]

## 5 Conclusion

In this paper, we used a machine learning method to predict the transition temperature of Fe-based superconductors based on the lattice parameters. By training BP algorithm, the acceptable accuracy of 91.181% was obtained in the model with available data. We made the performance measurement for estimating the model stability, and the model errors were in a controllable range. We used the trained model to predict, and the predictive value is close to the actual value. Those suggest that the model is capable of estimating the transition temperature of Fe-based superconductors with reasonable accuracy and therefore is recommended for predicting the transition temperature of Fe-based superconductors.
